# Supplementary material for: Evidence that the human cell cycle is a series of uncoupled, memoryless phases
Source: Mol Syst Biol. 2019 Mar 19;15(3):e8604. doi: 10.15252/msb.20188604 (PMC6423720; doi:10.15252/msb.20188604)
Supplement: Supplementary file 1 — Appendix [file MSB-15-e8604-s001.pdf]

Appendix for:

Evidence that the human cell cycle is a series of uncoupled, memoryless phases

Hui Xiao Chao[1,2], Randy I. Fakhreddin[1], Hristo K. Shimerov[1], Katarzyna M. Kedziora[1], Rashmi J. Kumar[1,3], Joanna Perez[4], Juanita C. Limas[5], Gavin D. Grant[4,6], Jeanette Gowen Cook[4,6], Gaorav P. Gupta[6,7], and Jeremy E. Purvis[1,2,3,6,†]

[1]Department of Genetics
[2]Cirriculum for Bioinformatics and Computational Biology
[3]Curriculum in Genetics and Molecular Biology
[4]Department of Biochemistry and Biophysics
[5]Department of Pharmacology
[6]Lineberger Comprehensive Cancer Center
[7]Department of Radiation Oncology

University of North Carolina at Chapel Hill
120 Mason Farm Road
Chapel Hill, NC 27599-7264

[†]Corresponding Author:
Jeremy Purvis
Genetic Medicine Building 5061, CB#7264
120 Mason Farm Road
Chapel Hill, NC 27599-7264

Table of Contents

Figure S1. The PCNA-mCherry reporter and imaging condition do not alter the cell cycle dynamics. 3

Figure S2. Pairwise correlations between cell cycle phases with automated phase detection and PIP-FUCCI reporter. 6

Figure S3. The quiescent (G0) population under proliferating conditions is low compared to other phases. 7

Figure S4. Pairwise correlations between cell cycle phases in three human cell lines. 9

Figure S5. Correlation among cell cycle phase durations between sibling cells and between adjacent mother daughter gap phases. 10

Figure S6. The Pearson correlation coefficient's dependence on sampling frequency. 12

Figure S7. Fitting with a single rate parameter for all phases is insufficient to recapitulate the cell cycle distribution. 14

Figure S8. Perturbing cell cycle phase durations and phase coupling in RPE cells. 16

Figure S9. Phase coupling with DNA damage perturbation. 17

Figure S10. Correlation in cell cycle phase durations and its origin. 18

Figure S11. A model for the heritable factors governing phase progression rate. 19

Figure S12. Bootstrap analysis for correlation coefficients between cell cycle phases under perturbation in U2OS. 21

Table S1. Correlation coefficient and significance with different linear regression methods. 23

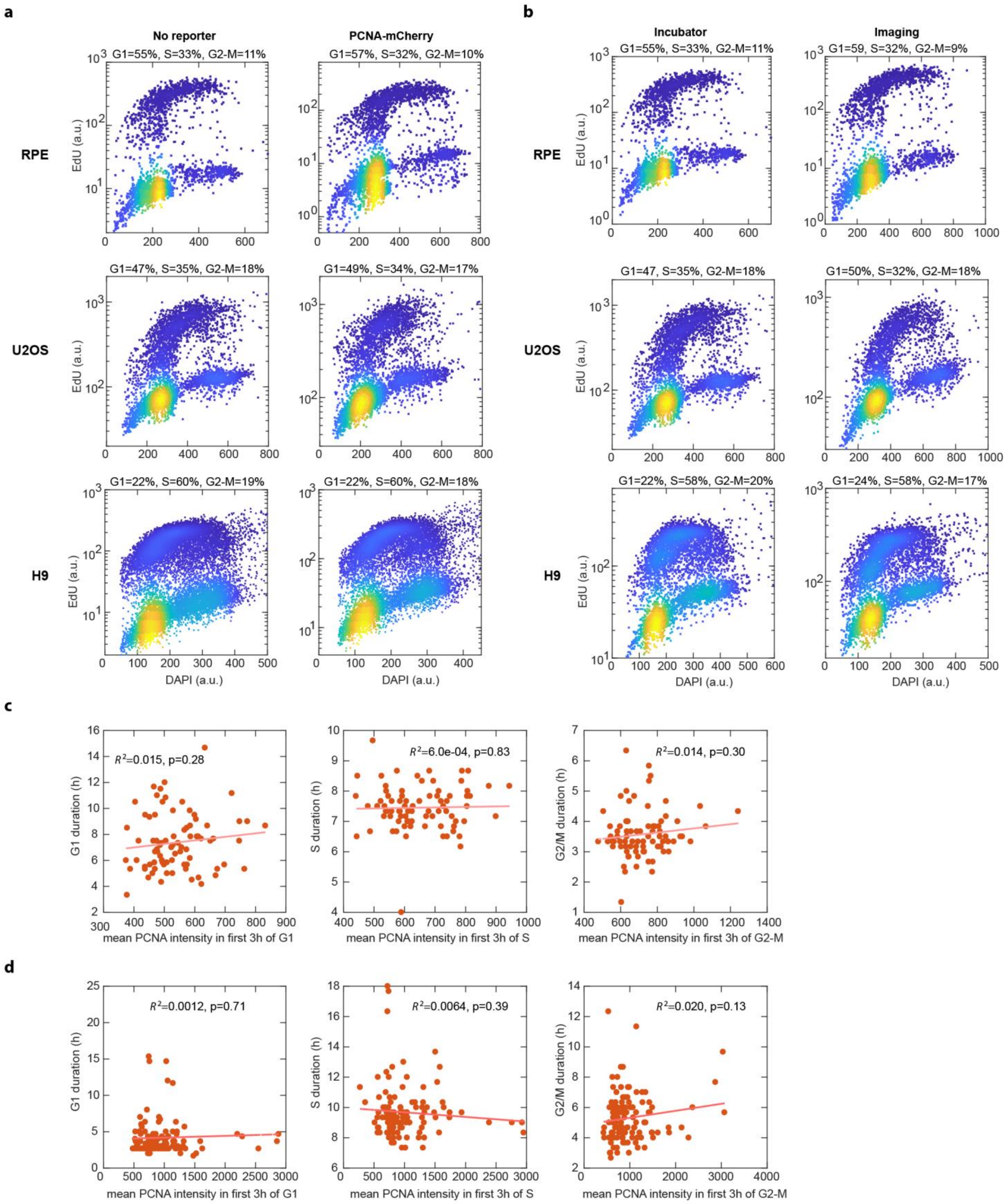

Figure S1. The PCNA-mCherry reporter and imaging condition do not alter the cell cycle dynamics. **a,** RPE, U2OS, and H9 cell lines with and without the PCNA-mCherry reporter were pulse with 30mins EdU and quantified for cell cycle phase distributions. n>3000 for each condition. **b**, RPE, U2OS, and H9 cell lines were imaged every 10 mins with the same imaging settings for acquiring phase duration data for 72 hours before 30min EdU pulse and quantification of cell cycle phase distributions. The control groups were growing inside

the incubator for 72 hours before the EdU pulse. n>3000 for each condition. **c**, For single RPE cells, the mean PCNA-mCherry intensity at the beginning of a phase (averaged among the first 3 hour) was plotted against the duration of that phase. p indicates p-value. n=80. **d**, Same as **Figure S1c**, but for U2OS. n=117.

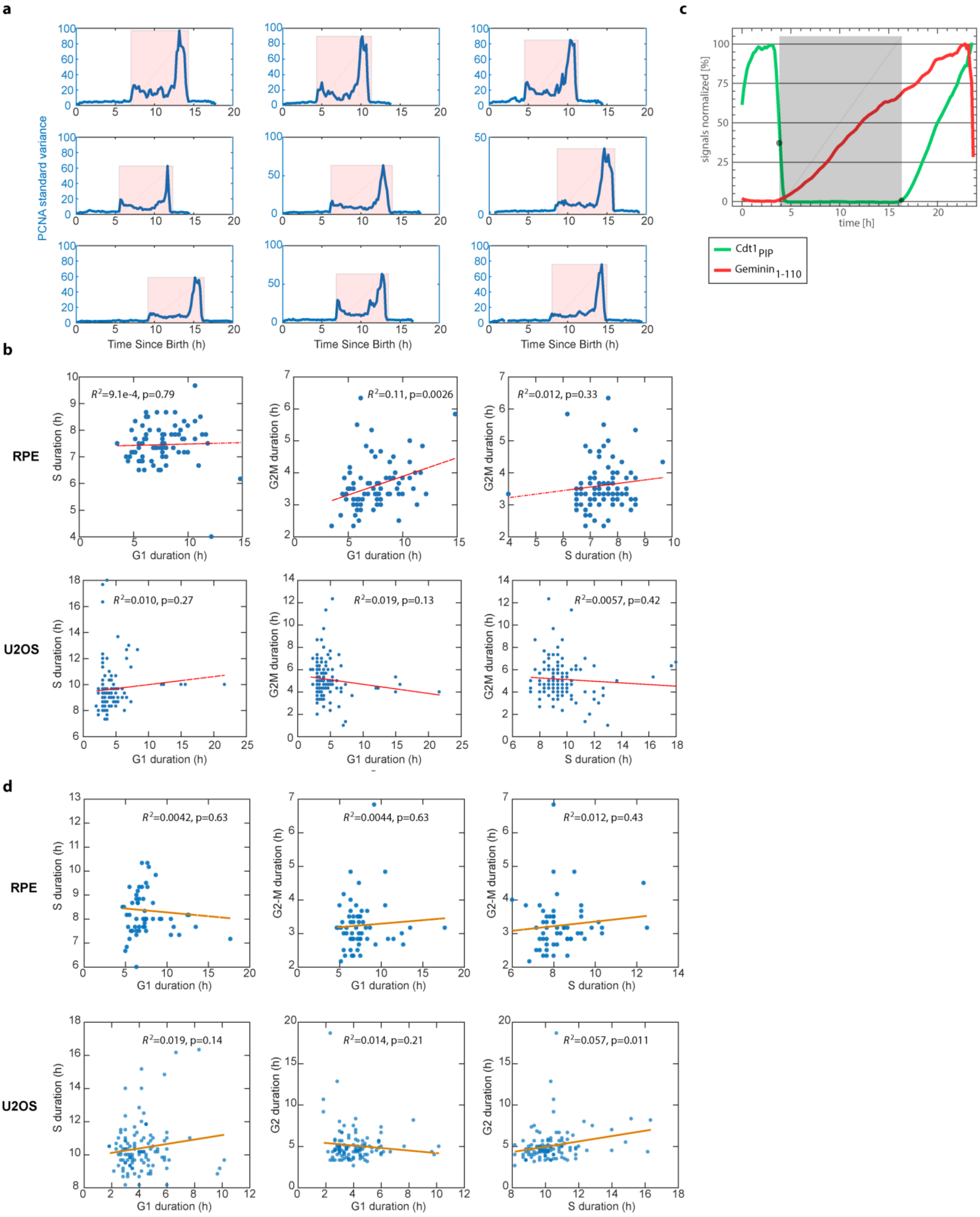
a
PCNA standard variance
Time Since Birth (h)
b
RPE
U2OS
S duration (h)
G2M duration (h)
G1 duration (h)
S duration (h)
R²=9.1e-4, p=0.79
R²=0.11, p=0.0026
R²=0.012, p=0.33
R²=0.010, p=0.27
R²=0.019, p=0.13
R²=0.0057, p=0.42
c
signals normalized [%]
time [h]
Cdt1PIP
Geminin1-110
d
RPE
U2OS
S duration (h)
G2-M duration (h)
G2 duration (h)
G1 duration (h)
S duration (h)
R²=0.0042, p=0.63
R²=0.0044, p=0.63
R²=0.012, p=0.43
R²=0.019, p=0.14
R²=0.014, p=0.21
R²=0.057, p=0.011

Figure S2. Pairwise correlations between cell cycle phases with automated phase detection and PIP-FUCCI reporter. **a,** Examples of single cell trajectories of PCNA standard variance (see supporting manuscript and Method Details). The shaded area represents S phase determined by the PCNA standard variance metric. **b**, Correlation between cell cycle phase durations determined by the PCNA standard variance shown in **Figure S2a**. p indicates p-value. RPE, n = 79; U2OS, n = 119. **c**, An example of a single cell trajectory of the PIP-FUCCI reporter (see Method Details). The shaded area represents S phase determined based on the Cdt1 mean intensity values (black circles). **d**, Correlation between cell cycle phase durations determined by the PIP-FUCCI reporter (see Method Details). p indicates p-value. RPE, n = 57; U2OS, n = 114.

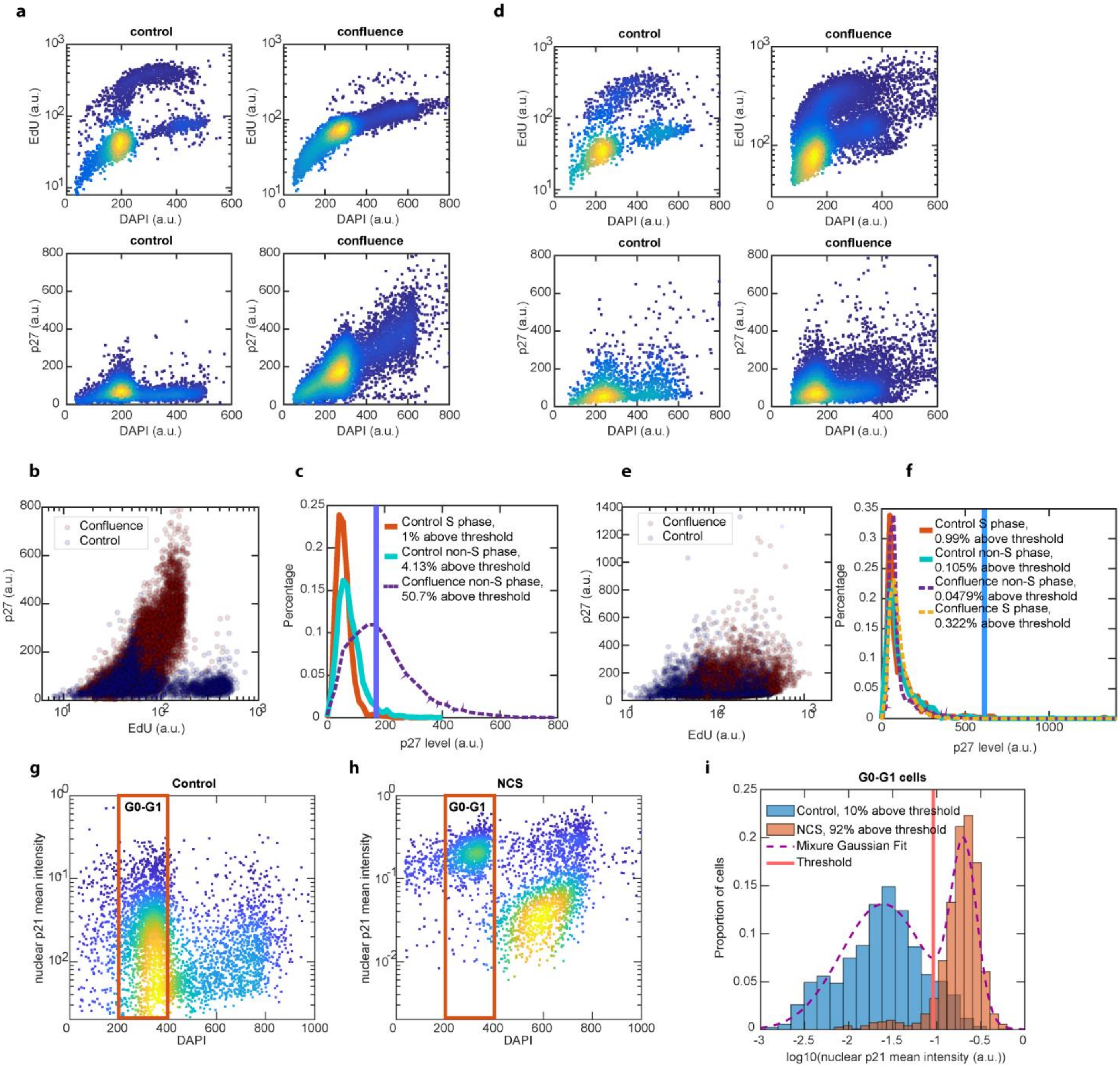

Figure S3. The quiescent (G0) population under proliferating conditions is low compared to other phases. **a**, Scatter plots of RPE cells with DAPI, EdU, and p27 quantification. For control, cells were seeded at 5% initial density and grown for 72 h before 30 mins of EdU pulse. For the confluence condition, cells were seeded at 80% initial density and grown for 72 h before 30 mins of EdU pulse. n = 4264 (control); n = 13580 (confluence). **b**, Scatter plot of p27 level in the confluence growing condition as a function of EdU incorporation level. **c,** Histograms of p27 expression levels. For the control population, cells were categorized into S phase and non-S phase based on the EdU level in panel a. A p27 threshold was calculated so that 1% of the S phase cells, which were presumably non-G0, were above the threshold. This same threshold value was then used to calculate the percentage of non-S phase control cells above this threshold, in both the control and the confluence conditions. A percentage for S phase cells in the confluence condition was not quantified because only a small portion of cells (<0.5%) were EdU positive. **d,** Same as panel a, but for U2OS. n = 1459 (control); n = 14467 (confluence). e, Same as panel b, but for U2OS. **f,** Same as panel c, but for U2OS. Here, the percentage of S phase cells in the confluence condition was quantified due to a large S phase population. **g,** Scatter plot of RPE cells with DAPI and p21 quantification. Box area are analyzed as G1 cells. n=4172. **h,**

Scatter plot of RPE cells treated with 100 ng/mL NCS. 13 hours post treatment, cells were stained for DAPI and p21 quantification. Box area are analyzed as G1 cells. n=3121. **i,** Histograms of nuclear mean p21 level of the G1 cells identified in **Figure S3g-h**. A Gaussian mixture model with two components was fitted to the data, and a threshold that separates high versus low p21 level was obtained by finding the p21 level where the probabilities of being in the high p21 and low p21 groups are equal.

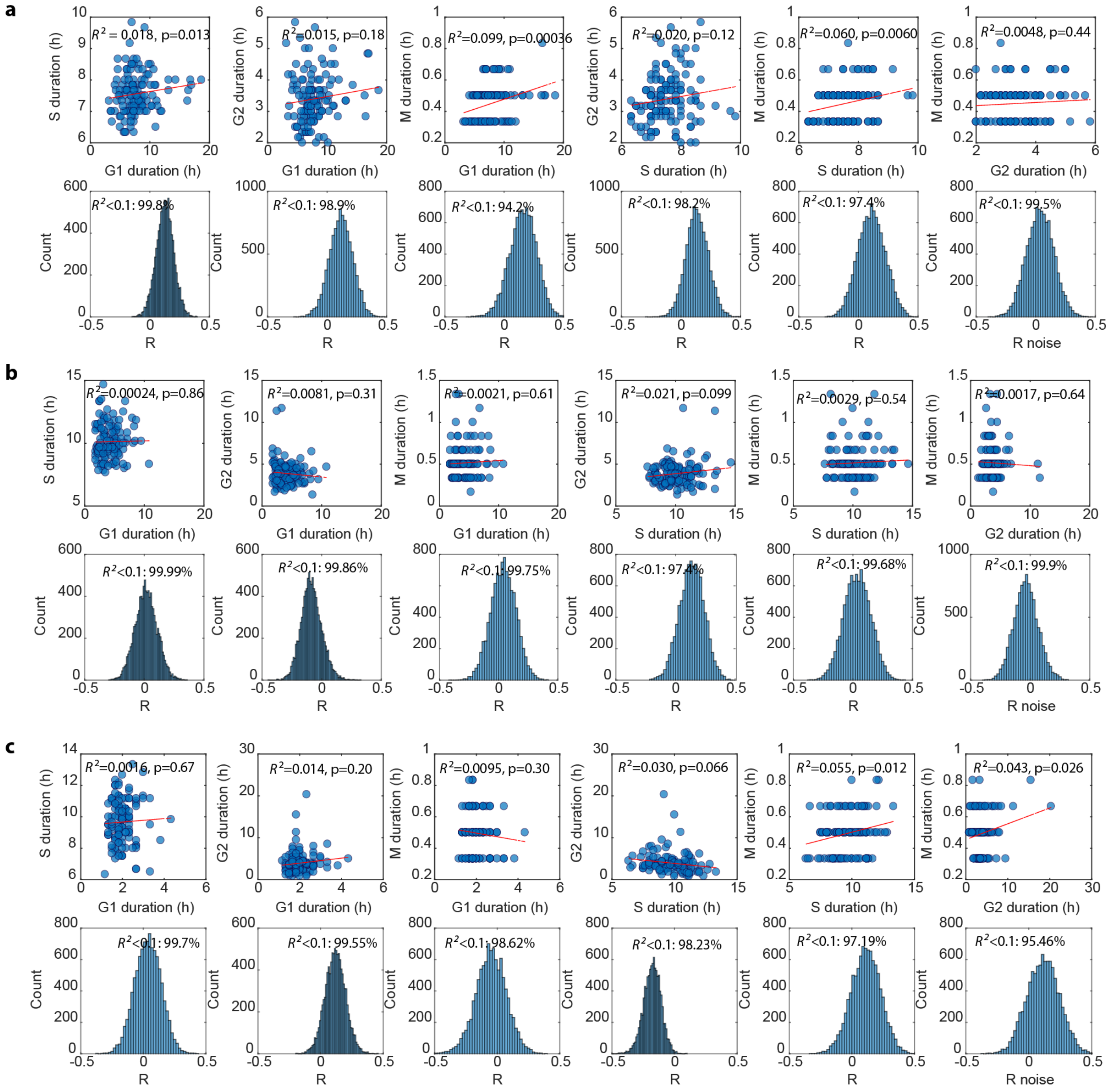

Figure S4. Pairwise correlations between cell cycle phases in three human cell lines. a, Upper panel: correlation between the cell cycle phase durations in RPE. Lower panel: Non-parametric bootstrap of the distribution of correlation coefficient (R), with consideration of measurement error (see Method details). p indicates p-value. n=10,000. **b,** Same as **Figure S4a**, but in U2OS. **c,** Same as **Figure S4a**, but in H9.

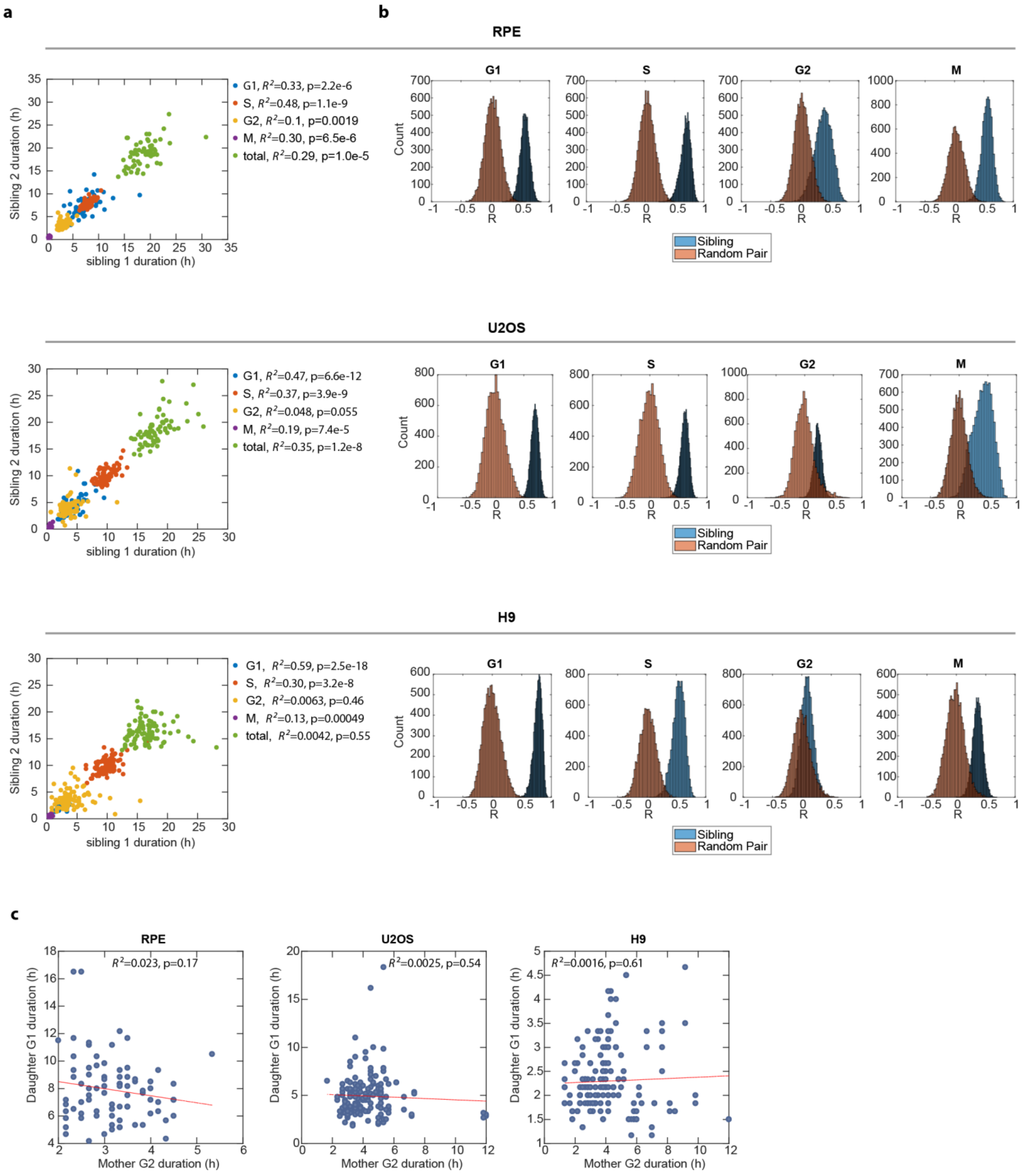

Figure S5. Correlation among cell cycle phase durations between sibling cells and between adjacent mother daughter gap phases. a, Correlation between the cell cycle phase durations of sibling cells in RPE (upper panel), U2OS (middle panel), and H9 (lower panel). Data were fit with linear regression, and Pearson correlation coefficients were calculated. p indicates p-value. n >117. **b**, Non-parametric bootstrap of the distribution of Pearson correlation coefficient (R) between sibling pairs in panel a. n =10,000. For the random control, two random cells were selected as pairs, and the number of pairs was chosen to match the number of

sibling pairs in panel a. **c**, Correlation between the G2 duration of a mother cell and the G1 duration of its daughter cell in RPE (left panel, n = 81), U2OS (middle panel, n = 148), and H9 (right panel, n = 165). p indicates p-value.

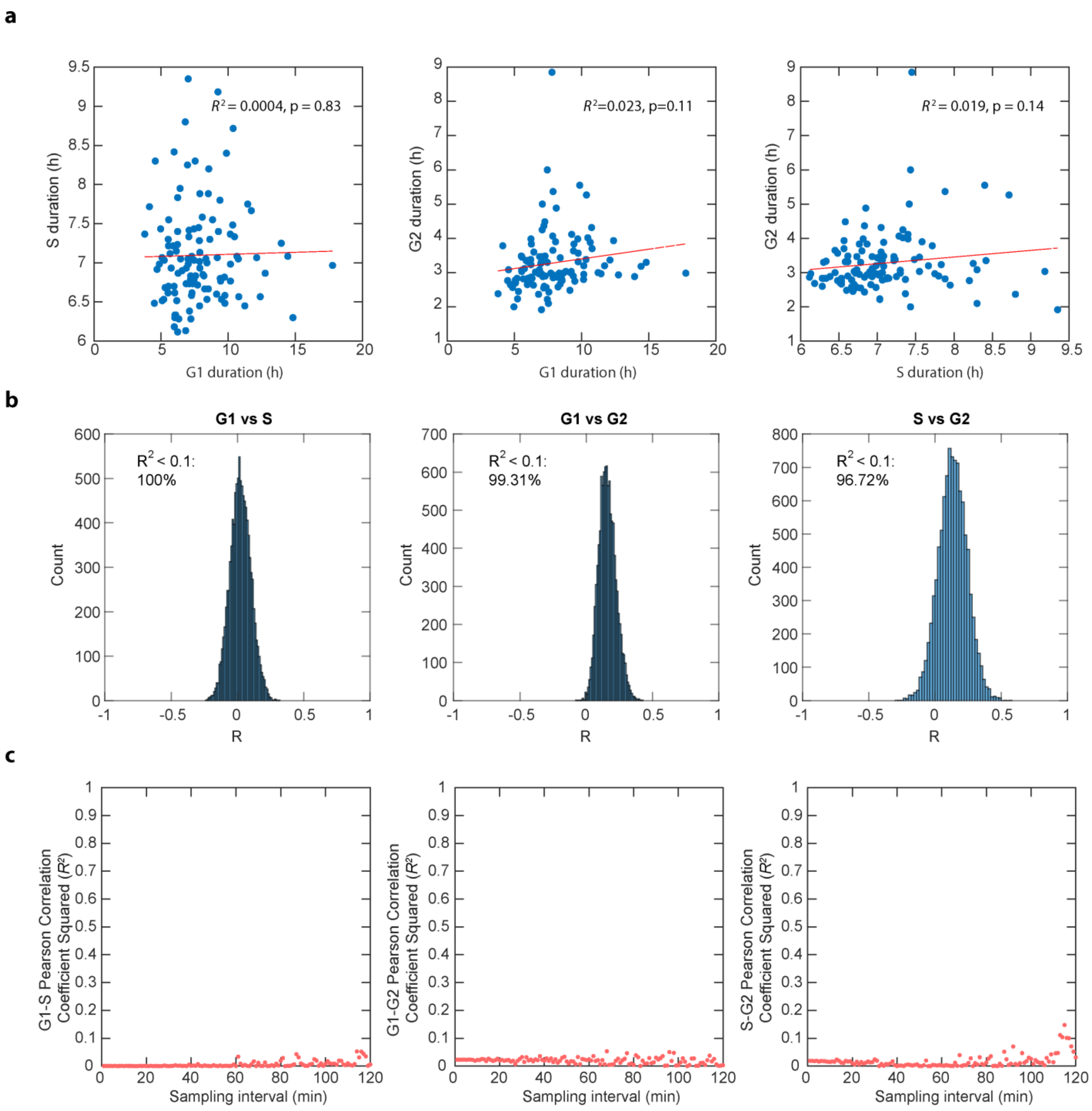

Figure S6. The Pearson correlation coefficient's dependence on sampling frequency. a, Correlations between individual cell cycle phase durations in RPE cells imaged at 1 minute interval. n = 113. R 2, square of Pearson correlation coefficient. p, p-value that the correlation is significant. **b,** Non-parametric bootstrap of the distribution of correlation coefficient (R). n=10,000. **c,** Pearson correlation coefficient squared as a function of sampling time interval. The effect of sampling at different time interval was reproduced by reading in the cell cycle phase transition time points based on different sampling time intervals. For each sampling rate, the R 2 was calculated and was plotted as a function of the sampling time interval.

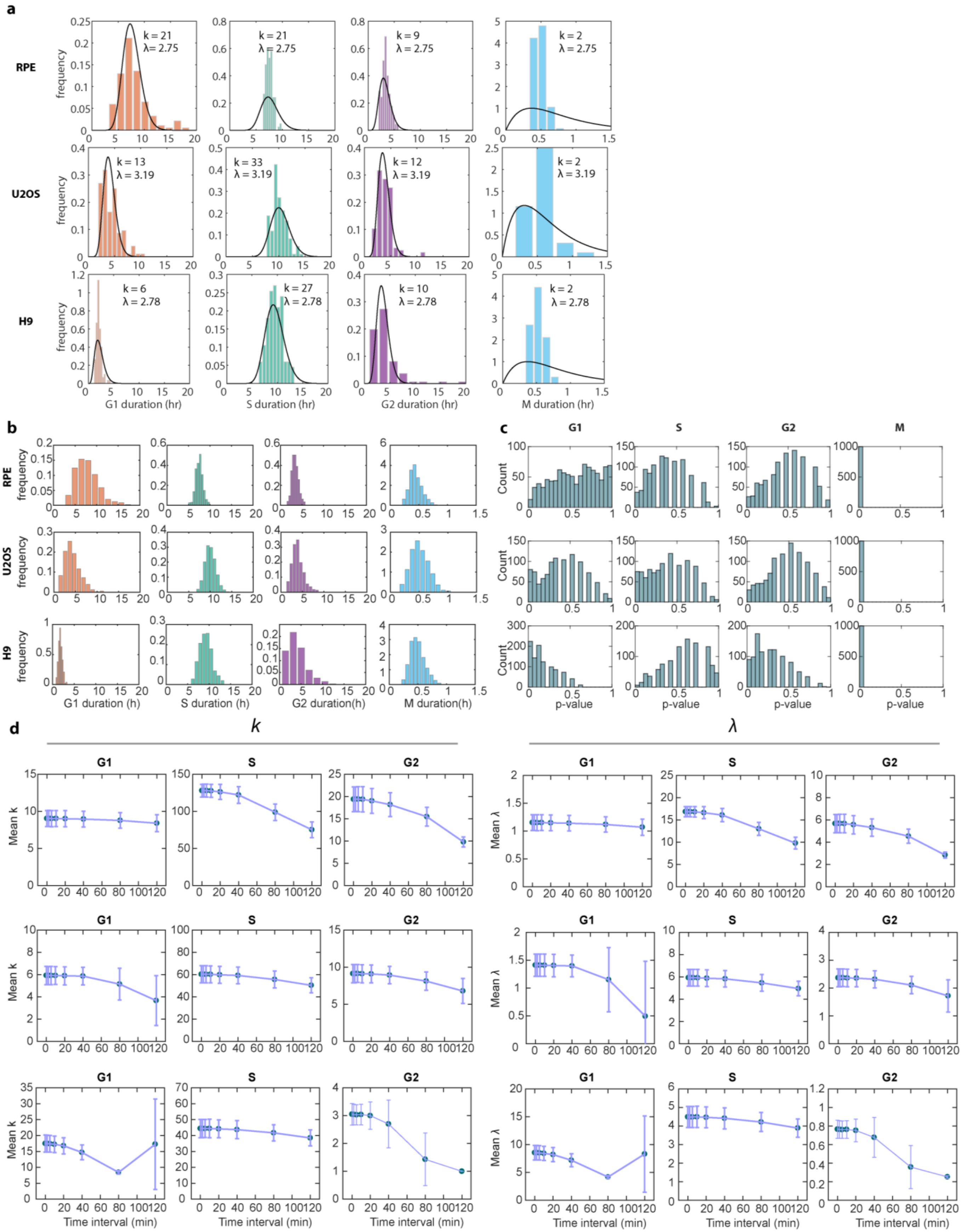

a
RPE
U2OS
H9
frequency
k = 21
λ = 2.75
k = 21
λ = 2.75
k = 9
λ = 2.75
k = 2
λ = 2.75
k = 13
λ = 3.19
k = 33
λ = 3.19
k = 12
λ = 3.19
k = 2
λ = 3.19
k = 6
λ = 2.78
k = 27
λ = 2.78
k = 10
λ = 2.78
k = 2
λ = 2.78
G1 duration (hr)
S duration (hr)
G2 duration (hr)
M duration (hr)
b
G1 duration (h)
S duration (h)
G2 duration(h)
M duration(h)
c
G1
S
G2
M
Count
p-value
d
k
λ
Mean k
Mean λ
Time interval (min)

Figure S7. Fitting with a single rate parameter for all phases is insufficient to recapitulate the cell cycle distribution. **a,** Distributions of cell cycle phase durations fitted with a simple Markovian model with a single rate parameter. **b,** Simulations of the cell cycle phase durations of the Erlang model based on the fitted parameters. **c,** Distribution of the p-value, based on the Kolmogorov–Smirnov test, for significant difference in the cell cycle phase distributions between the experimental data and the simulated data from the Erlang model. For each simulation, 200 cells were generated, with a total of 1000 simulations. **d,** Erlang parameter dependence on sampling time interval. Cell cycle phase durations were simulated using the the Erlang parameters with the same cell number as the experimental data. The sampling effect was reproduced by binning the data into different intervals, and the data was fitted with an Erlang distribution. This process was iterated 100 times to obtain the mean Erlang parameters and their standard deviations (errorbars) as a function of sampling time.

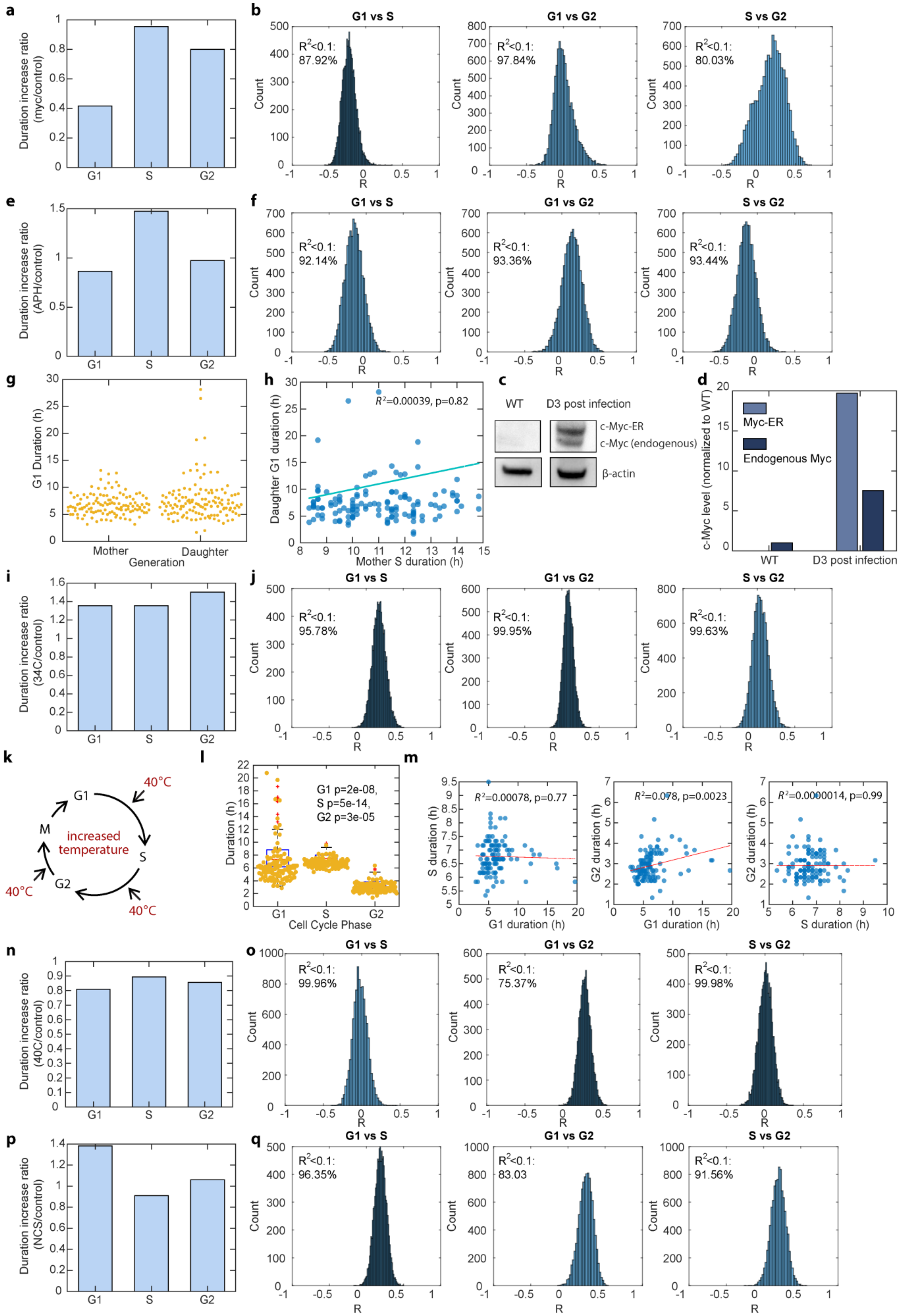
a
Duration increase ratio (myc/control)
G1
S
G2
b
G1 vs S
R²<0.1: 87.92%
G1 vs G2
R²<0.1: 97.84%
S vs G2
R²<0.1: 80.03%
Count
R
e
Duration increase ratio (APH/control)
f
G1 vs S
R²<0.1: 92.14%
G1 vs G2
R²<0.1: 93.36%
S vs G2
R²<0.1: 93.44%
g
G1 Duration (h)
Mother
Daughter
Generation
h
R²=0.00039, p=0.82
Daughter G1 duration (h)
Mother S duration (h)
c
WT
D3 post infection
c-Myc-ER
c-Myc (endogenous)
β-actin
d
Myc-ER
Endogenous Myc
c-Myc level (normalized to WT)
WT
D3 post infection
i
Duration increase ratio (34C/control)
j
G1 vs S
R²<0.1: 95.78%
G1 vs G2
R²<0.1: 99.95%
S vs G2
R²<0.1: 99.63%
k
G1
S
G2
M
40°C
40°C
40°C
increased temperature
l
G1 p=2e-08,
S p=5e-14,
G2 p=3e-05
Duration (h)
Cell Cycle Phase
m
R²=0.00078, p=0.77
S duration (h)
G1 duration (h)
R²=0.078, p=0.0023
G2 duration (h)
G1 duration (h)
R²=0.0000014, p=0.99
G2 duration (h)
S duration (h)
n
Duration increase ratio (40C/control)
o
G1 vs S
R²<0.1: 99.96%
G1 vs G2
R²<0.1: 75.37%
S vs G2
R²<0.1: 99.98%
p
Duration increase ratio (NCS/control)
q
G1 vs S
R²<0.1: 96.35%
G1 vs G2
R²<0.1: 83.03
S vs G2
R²<0.1: 91.56%

Figure S8. Perturbing cell cycle phase durations and phase coupling in RPE cells. a, Ratio of RPE's cell cycle phase duration increase with myc overexpression relative to control. **b,** Non-parametric bootstrap of the distribution of correlation coefficient (R) for RPE under myc overexpression. n=10,000. **c,** Western blot for overexpression of myc in RPE cells transfected with retroviral Myc-ER on day 3 (D3) post infection. WT represents wildtype RPE cells without the Myc-ER construct and transfection. **d,** Quantification of Myc signal from **Figure S8c**. **e,** Ratio of RPE's cell cycle phase duration increase under 50 ng/mL APH treatment relative to control. **f,** Non-parametric bootstrap of the distribution of correlation coefficient (R) for RPE under 50 ng/mL APH. n=10,000. **g,** G1 durations in the mother cells (treated during S phase) and in the daughter cells whose mothers were treated. p-value=0.0017 based on 2-sided Kolmogorov–Smirnov test. n>122. **h,** Correlation between S phase in the treated mother cells and the G1 duration in the daughter cells. Data were fitted with linear regression and Pearson correlation coefficient. n=130. **i,** Ratio of RPE's cell cycle phase duration increase at 34°C relative to control (37°C). **j,** Non-parametric bootstrap of the distribution of correlation coefficient (R) for RPE at 34°C. n=10,000. **k,** Schematic of shortening all phases by incubating cells at 40°C. **l,** Shift in phase durations of RPE cells incubated at 40°C. **m,** Pairwise correlation between phase durations for cells incubated at 40°C. n=114. **n,** Ratio of RPE's cell cycle phase duration increase at 40°C relative to control (37°C). **o,** Non-parametric bootstrap of the distribution of correlation coefficient (R) for RPE at 40°C. n=10,000. **p,** Ratio of RPE's cell cycle phase duration increase under 25 ng/mL NCS treatment relative to control. **q,** Non-parametric bootstrap of the distribution of correlation coefficient (R) for RPE under 25 ng/mL NCS treatment. n=10,000.

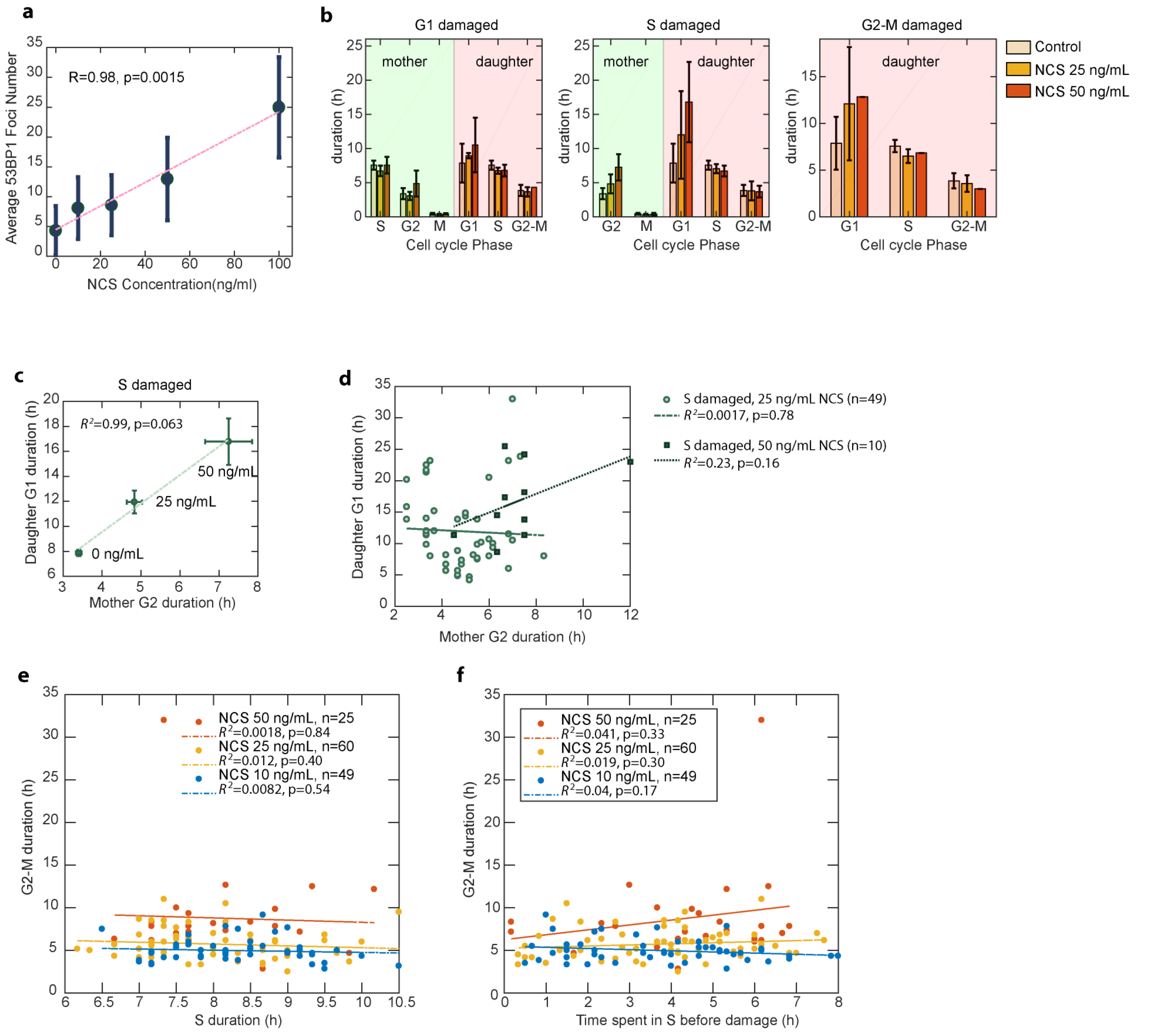

Figure S9. Phase coupling with DNA damage perturbation. **a,** Mean 53BP1 foci number as a function of NCS concentration. RPE cells were treated with NCS and then quantified for 53BP1 foci number 1 hour post treatment. Error bars represent standard deviations. **b,** Cell cycle phase durations of RPE cells whose mother cells were treated with NCS during G1 (left panel), S (middle panel), or G2-M (right panel) phases. The cell cycle phase durations of the subsequent cell cycle of the daughter cells were also measured. Error bar represents standard deviation. **c,** Correlation between the population mean G2 duration of the mother cell treated in S phase and the mean G1 durations of their daughter cells, grouped by different NCS concentrations, fitted with linear regression. Error bars represent standard error of mean. **d,** Correlation between the G2 durations of the mother cell treated in S phase and the G1 durations of the daughter cells, fitted with linear regression. Legend indicates the phase in which the mother cell was damaged at the indicated NCS concentrations. **e,** Correlation between S and G2-M durations in RPE cells damaged during their S phase with different NCS concentrations. **f,** Duration of G2-M as a function time spent in S phase before NCS treatment. Same data points as in **Figure S9e**.

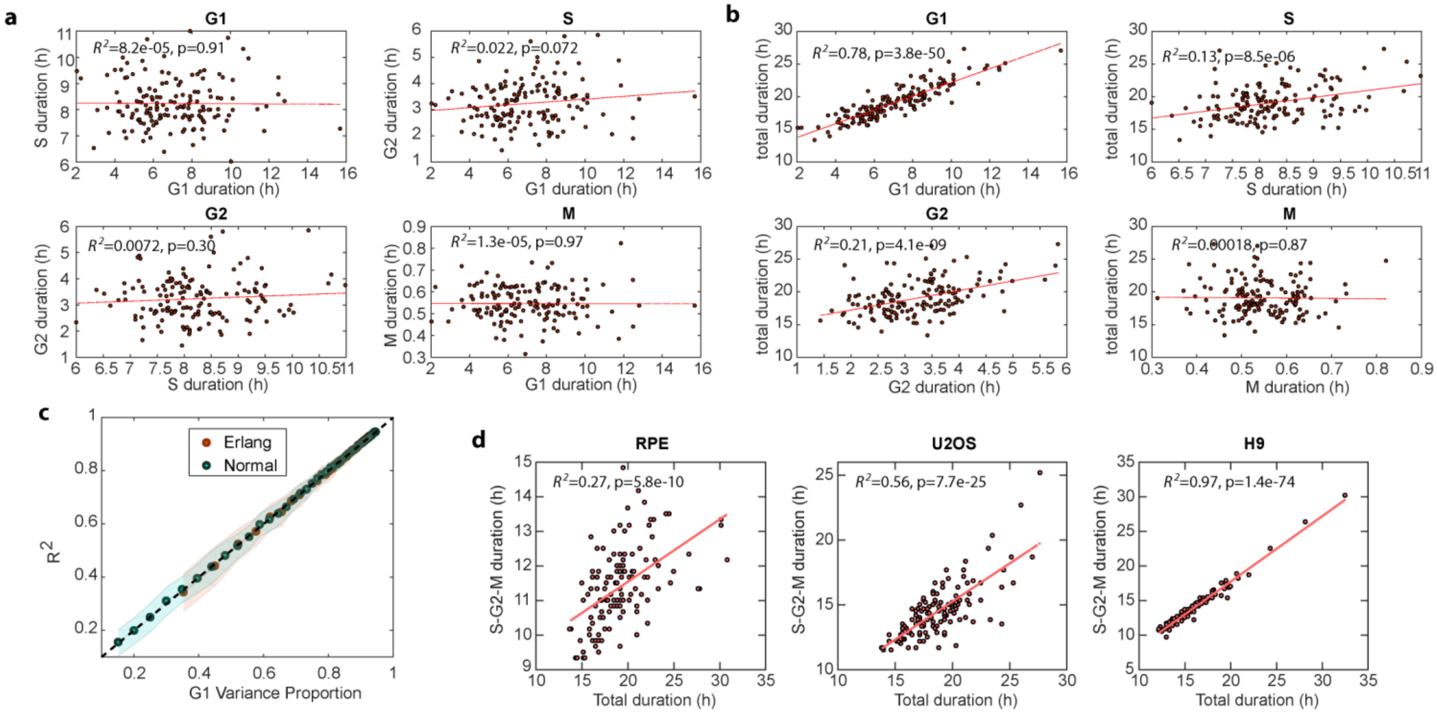

Figure S10. Correlation in cell cycle phase durations and its origin. a, Simulation of pairwise correlation between cell cycle phase durations under the Erlang model. The parameters were from the fitted RPE cell data in **Figure 2a**. **b,** Simulation of correlation between each cell cycle phase and the total cell cycle durations under the Erlang model, as **in Figure S10a**. **c**, Simulation of correlation coefficients as a function of the variance in total cell cycle duration contributed by G1. Data were simulated either under the Erlang model, as in **Figure S10a-b**, or under the normal distribution model. For the normal distribution model, parameters were chosen according to the mean and variance of the cell cycle phase duration's distributions. The dashed line represents the diagonal line. **d,** Correlation between the combined S-G2-M phase and the total cell cycle duration in RPE (left), U2OS (middle), and H9 (right) cells' experimental data. Data were fitted with linear regression.

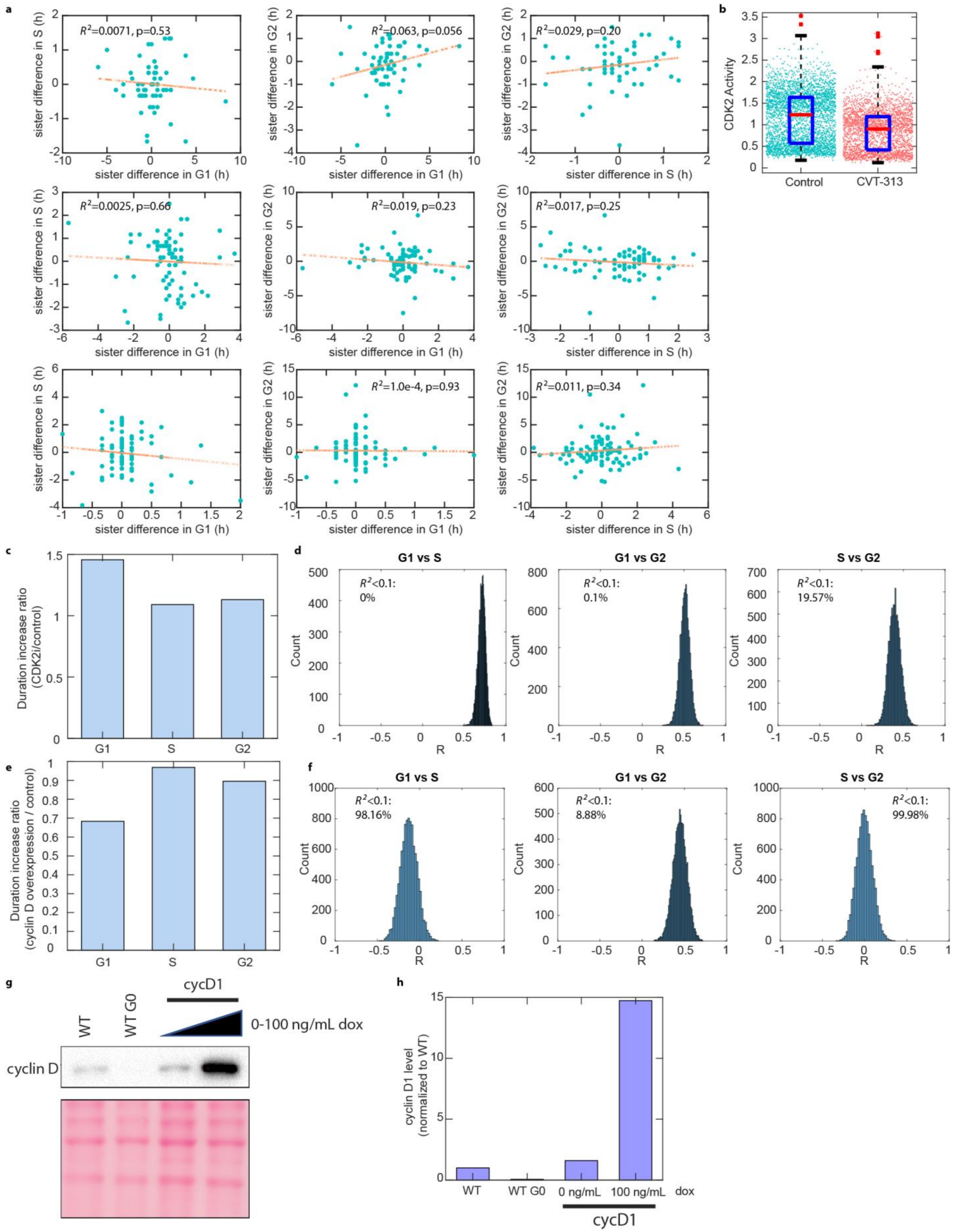

Figure S11. A model for the heritable factors governing phase progression rate. **a,** Correlation plots in the difference in cell cycle phase durations between the sibling cells. To calculate the difference, the

subtrahend and minuend were randomly chosen between the sibling cells. n>108. **b,** CDK2 activity upon 2μM CVT-313 treatment. CDK2 activity was quantified 1 hour post treatment using the DHB-mCherry reporter as the ratio of cytoplasmic versus nuclear mean intensity. p-value= 3e-135 based on 2-sided Kolmogorov–Smirnov test. of cell cycle phase duration increase with CDK2 inhibitor treatment relative to control. **c,** Ratio of cell cycle phase duration increase with CDK2 inhibitor treatment relative to control. **d,** Non-parametric bootstrap of the distribution of correlation coefficient (R) for RPE under CDK2 inhibitor treatment. n=10,000. **e,** Ratio of RPE's cell cycle phase duration decrease with cyclin D overexpression relative to control. **f,** Non-parametric bootstrap of the distribution of correlation coefficient (R) for RPE with cyclin D overexpression. n=10,000. **g,** Western blot for expression of dox-inducible cyclin D1 in RPE cells 5 hours after 100 ng/mL doxycycline addition. WT represents parent RPE cells without the cyclin D1 construct. WT G0 are cells rendered quiescent by contact inhibition. **h,** Quantification of cyclin D1 levels from **Figure S11g**.

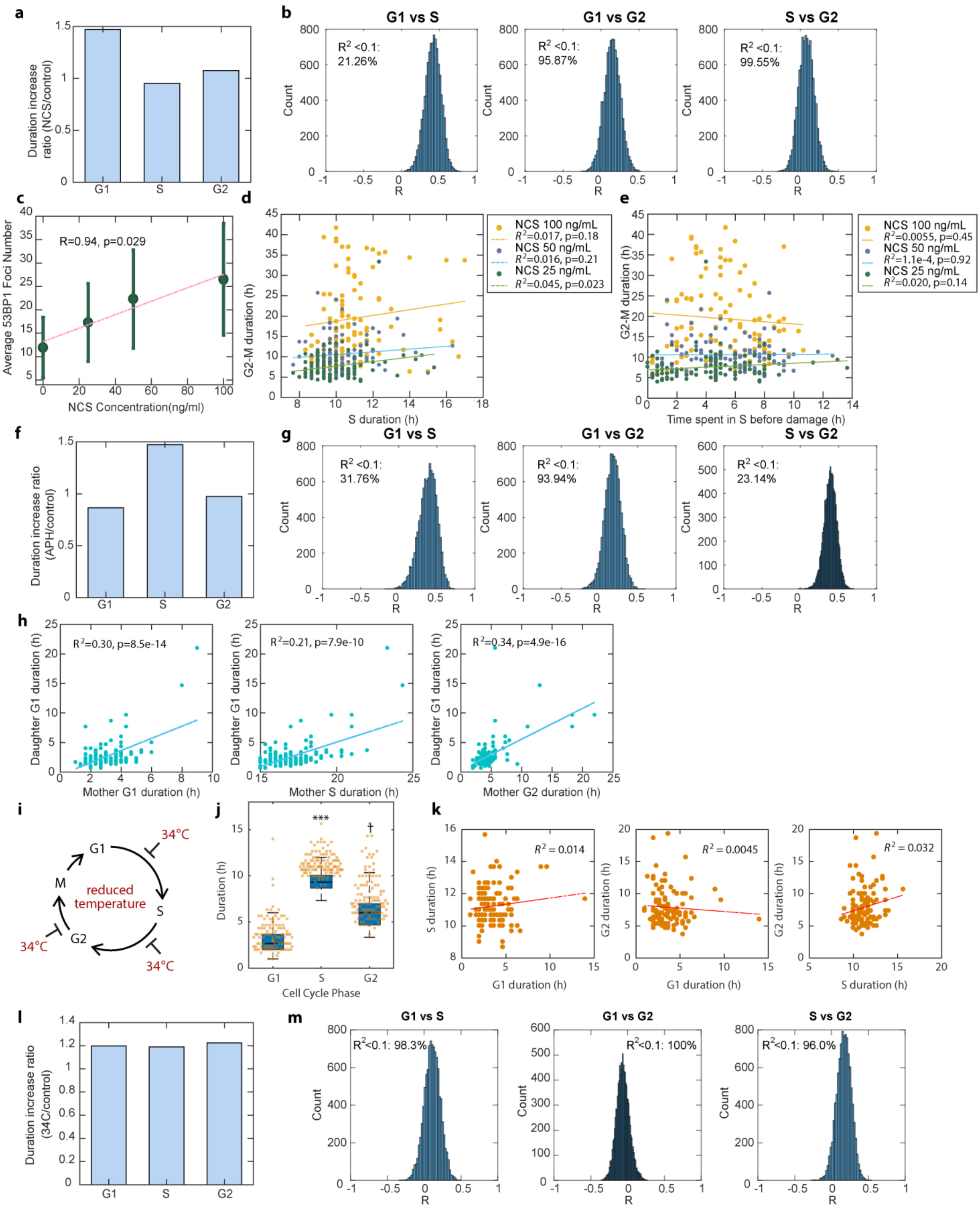

Figure S12. Bootstrap analysis for correlation coefficients between cell cycle phases under perturbation in U2OS. **a,** Ratio of U2OS's cell cycle phase duration increase under 100 ng/mL NCS treatment relative to control. **b,** Non-parametric bootstrap of the distribution of correlation coefficient (R) for U2OS under 100 ng/mL NCS treatment. n=10,000. **c,** Mean 53BP1 foci number as a function of NCS concentration. U2OS

cells were treated with NCS and then quantified for 53BP1 foci number 1 hour post treatment. Error bars represent standard deviations. **d,** Correlation between S and G2-M durations in RPE cells damaged during their S phase with different NCS concentrations. n=106 (100 ng/mL); 97 (50 ng/mL); 114 (25 ng/mL). **e,** Duration of G2-M as a function time spent in S phase before NCS treatment. Same data points as in **Figure S12d**. **f,** Ratio of U2OS's cell cycle phase duration increase under 50 ng/mL APH treatment relative to control. **g,** Non-parametric bootstrap of the distribution of correlation coefficient (R) for U2OS under 50 ng/mL APH treatment. n=10,000. **h,** Correlation between phases in the treated mother cells and the G1 duration in the daughter cells. Data were fitted with linear regression and Pearson correlation coefficient. n=160. **i,** Schematic of prolonging all phases by growing under 34°C condition. **j,** Cell cycle phase durations of U2OS cells growing under 34°C condition. Boxplots for untreated cells are underlaid for comparison. ***, P < 1 × 10-20, †, P < 1 × 10-4, 2-sided Kolmogorov–Smirnov test. n = 112. **k,** Pairwise correlation between cell cycle phase durations growing under 34°C condition. **l** Ratio of U2OS's cell cycle phase duration increase under 34°C growing condition relative to control. **m,** Non-parametric bootstrap of the distribution of correlation coefficient (R) for U2OS under 34°C growing condition. n = 10,000.

Table S1. Correlation coefficient and significance with different linear regression methods.
For the winsorization, the threshold for replacing extreme values was set to be 90% percentile.

		G1-S		G1-G2		S-G2	
		R^2	p-value	R^2	p-value	R^2	p-value
**RPE**	Pearson	0.018396388	0.131511	0.014593	0.179606	0.019715	0.118329
	Spearman	0.026126627	0.071724	0.015156	0.17138	0.028598	0.059386
	Kendall	0.0122576	0.080145	0.009354	0.124062	0.015667	0.051784
	Winsorize	0.019076453	0.124524	0.018392	0.131561	0.029769	0.054341
**U2OS**	Pearson	0.000235173	0.862517	0.008148	0.307098	0.021102	0.099137
	Spearman	0.003063314	0.531682	0.004096	0.469425	0.001626	0.648707
	Kendall	0.001401575	0.542256	0.00213	0.452869	0.000493	0.719344
	Winsorize	0.001883244	0.623959	0.003629	0.495962	0.006931	0.346361
**H9**	Pearson	0.001664173	0.667919	0.014255	0.207822	0.029764	0.067659
	Spearman	0.003949215	0.508449	0.029494	0.068937	0.060204	0.008808
	Kendall	0.002123751	0.498697	0.014624	0.074802	0.027	0.012255
	Winsorize	0.004073638	0.501831	0.02737	0.079915	0.059337	0.009324
**RPE myc**	Pearson	0.045586408	0.021376	6.22E-05	0.933023	0.047445	0.018832
	Spearman	0.01522929	0.186905	0.005023	0.449647	0.006258	0.398599
	Kendall	0.009054649	0.155221	0.003292	0.400271	0.004017	0.355278
	Winsorize	0.040899672	0.029472	0.003386	0.534937	0.001066	0.727937
**RPE APH**	Pearson	8.98E-05	0.921388	0.023516	0.108083	0.004618	0.478536
	Spearman	0.000285445	0.860295	0.025254	0.09573	0.000114	0.911623
	Kendall	4.55E-07	0.993887	0.012215	0.099755	0.000129	0.867825
	Winsorize	3.14E-06	0.985271	0.029235	0.072773	0.003718	0.524925
**RPE NCS**	Pearson	0.044460945	0.021344	0.0625	0.006105	0.05012	0.014383
	Spearman	0.078839803	0.001981	0.069637	0.003733	0.072882	0.002985
	Kendall	0.039573666	0.002327	0.029899	0.006998	0.038655	0.003276
	Winsorize	0.052398266	0.012278	0.058168	0.008234	0.07124	0.003343
**RPE 34°C**	Pearson	0.0292764	0.059516	0.007609	0.339372	0.001378	0.68484
	Spearman	0.08950409	0.000816	0.015003	0.178935	0.044476	0.019716
	Kendall	0.042292811	0.001176	0.007078	0.183117	0.022788	0.018586
	Winsorize	0.065214203	0.004528	0.016169	0.162801	0.024572	0.084658
**RPE 40°C**	Pearson	0.00079779	0.765465	0.0797	0.002346	1.486e-06	0.98973
	Spearman	0.025933	0.08696	0.134	6.05E-05	0.00263	0.587579
	Kendall	0.01101	0.115943	0.0747	4.14E-05	0.00127	0.603349
	Winsorize	0.000697	0.780356	0.141	3.97E-05	0.000186	0.885521
**RPE CDK2i**	Pearson	0.50383879	4.66E-19	0.260429	4.86E-09	0.159107	9.20E-06
	Spearman	0.548928669	1.96E-21	0.308216	9.96E-11	0.254287	7.87E-09
	Kendall	0.304128537	1.51E-17	0.160858	5.32E-10	0.137864	1.75E-08
	Winsorize	0.5265358	3.16E-20	0.28022	1.00E-09	0.223207	8.58E-08
**RPE cyclin D**	Pearson	0.0182	0.154363	0.2136	2.55E-07	8.69e-06	0.975278
	Spearman	0.0306	0.063683	0.2199	1.62E-07	0.00361	0.527257
	Kendall	0.0140	0.072354	0.1119	3.59E-07	0.00194	0.509096
	Winsorize	0.0245	0.097648	0.2309	7.21E-08	0.000979	0.742135
**U2OS APH**	Pearson	0.17949264	4.85E-08	0.035147	0.020311	0.166586	1.63E-07
	Spearman	0.046496337	0.007431	0.000306	0.830108	0.156543	4.15E-07
	Kendall	0.025205796	0.006684	0.000108	0.860868	0.084375	5.62E-07
	Winsorize	0.06339952	0.001691	0.003674	0.456692	0.14081	1.76E-06
**U2OS NCS**	Pearson	0.191921107	1.09E-06	0.022894	0.108056	0.006505	0.39362
	Spearman	0.150788094	1.96E-05	0.031018	0.060878	0.011085	0.264922
	Kendall	0.080804958	2.47E-05	0.016523	0.052833	0.004923	0.302318
	Winsorize	0.176115054	3.35E-06	0.022542	0.110834	0.009307	0.307219
**U2OS 34°C**	Pearson	0.014155232	0.211505	0.004527	0.480911	0.032024	0.059041
	Spearman	0.002642636	0.590378	0.006642	0.392963	0.030639	0.064901
	Kendall	0.001375721	0.591809	0.003799	0.362389	0.016907	0.056233
	Winsorize	0.007528012	0.363012	0.005084	0.455009	0.035819	0.045654
